# Statin treatment is not associated with an increased risk of adrenal insufficiency in real-world setting

**DOI:** 10.3389/fendo.2023.1254221

**Published:** 2023-09-25

**Authors:** Sandy Maumus-Robert, Ana Jarne-Munoz, Antoine Pariente, Thomas Duroux, Lise Duranteau, Julien Bezin

**Affiliations:** ^1^ University Bordeaux, INSERM, Bordeaux Population Health (BPH), Team AHeaD, Bordeaux, France; ^2^ CHU de Bordeaux, Clinical Pharmacology Unit, INSERM, Bordeaux, France; ^3^ Health Information Department, ELSAN Group, Paris, France; ^4^ Medical Gynaecology Department, AP-HP Paris Saclay University, Bicêtre Hospital, Paris, France

**Keywords:** statins, adrenal insufficiency, risk, pharmacoepidemiology, database

## Abstract

**Introduction:**

Statins could reduce the synthesis of steroid hormones, thereby could cause adrenal insufficiency. We investigated this risk in a large nationwide database.

**Methods:**

We conducted a nested case-control study using a cohort of individuals affiliated to the French health insurance system in 2010, ≥18y and without adrenal insufficiency history. Each case had a first event of adrenal insufficiency between 2015 and 2017 and was matched to up to ten controls on age, sex, and prior treatment with corticosteroids. Statin exposure was measured over the five years preceding the index date, considering a six-month censoring lag-time. Association was estimated using a conditional logistic regression adjusted for confounders included in a disease risk score. Analyses were stratified on age, sex and corticosteroid history of use.

**Results:**

4 492 cases of adrenal insufficiency were compared with 44 798 controls (median age 66y, 58% women), of which 39% vs. 33% were exposed to statins, respectively. No association between statin use and adrenal insufficiency was found when adjusting the model for confounders (adjusted odds ratio 0.98; 95% confidence interval 0.90-1.05). These results were consistent regardless of the exposure definition and stratifications considered.

**Conclusion:**

Statin-related adrenal insufficiency risk, if any, seems to be very limited and does not compromise the benefit of statin treatment.

## 1 Introduction

Statins are lipid-lowering drugs used to prevent cardiovascular diseases. They act by inhibiting the catalysis of 3-hydroxy-3-methylglutaryl coenzyme A (HMG CoA) into mevalonate by HMG CoA reductase, thus reducing cholesterol synthesis by the liver. Beside this effect on cholesterol reduction, statins reduce inflammation and oxidative stress and up-regulate endothelial NO-synthase activity, thereby improving endothelial functions and stabilizing the atherosclerotic plaque (1). Benefit of statins have been shown for both secondary and primary cardiovascular prevention (2–4). Statins are among the most commonly prescribed drugs worldwide (5). Around 200 million people take a daily statin worldwide, including over 30 million people in the United States alone (6). In France, about 5 million people were using such drugs in 2014 (7).

Statins have already been shown to increase the risk of diabetes through endocrine mechanisms related to HMG-CoA reductase activity (2, 8, 9). Steroid hormones are produced from cholesterol of low-density lipoproteins, which is used as a substrate of steroidogenesis in adrenal cortex and gonads (10, 11). By inhibiting cholesterol synthesis, statins could be responsible of a reduction of steroid hormones synthesis and thereby cause adrenal insufficiency (AI). Today, only a few studies concerning a limited number of patients showed contradictory results on steroid hormone synthesis (12–14).

In this context, we conducted a large, nationwide study based on French claims data to investigate if the use of statin is associated to occurrence of AI.

## 2 Methods

### 2.1 Data source

This study used the nationwide database of the French health care insurance system, SNDS (*Système National des Données de Santé*). SNDS provides individual and anonymous information for almost all French population (99%). It contains all outpatient healthcare reimbursements, including drugs, sociodemographic data (age, sex, social deprivation index based on geographic area, date of death), and all hospitalization discharge reports (dates, diagnoses, and performed procedures). For each reimbursed drug, data collected in the database include date of dispensing, active ingredients, route of administration, dosage, packaging, but not the prescribed daily dosage. Details on the French medico-administrative databases have been described elsewhere (15).

This study focused on the beneficiaries of the major health insurance scheme for employees (salaried workers and their relatives, retired salaried workers and their relatives); that is, 77% of the French population for whom the SNDS database has comprehensively recorded data since 2006.

### 2.2 Study design and participants

We conducted a nested case-control study. The source cohort was constituted by all individuals affiliated on January 1^st^ 2010 to the major health insurance scheme for at least three years before this date, aged 18 years or older in 2010, with no history of primary or secondary AI in the three years preceding baseline (without any hospitalization presenting ICD-10 diagnosis codes E271-E274 and without any use of hydrocortisone or desoxycortone identified by their respective ATC codes H02AB09 and H02AA03). Participants with AI diagnosis between January 1^st^ 2010 and December 31^st^ 2014 or with less than 5 years of follow-up were excluded from source cohort. Participants were followed until study outcome occurrence, December 31^st^ 2017, lost to follow-up or death, whichever came first.

### 2.3 Identification of cases

Incident cases of AI were identified from January 1^st^ 2015 to December 31^st^ 2017. It was defined as the presence of both a first hospital stay with a diagnosis of AI (ICD-10 code E271 to E274), and a first dispensing of hydrocortisone up to 30 days before or after hospital admission date. The index date was hospital admission date or hydrocortisone dispensing date, whichever came first.

### 2.4 Identification of controls

For each case was established a risk set of potential controls. Any subject included in the source cohort could be a potential control until the index date of the corresponding case. Each case was matched to up to ten controls using the Risk Set Sampling method. Matching criteria were age at index date (± two years), sex, and prior treatment with corticosteroids in the three years before the index date. Corticosteroid treatment history was assessed separately for each administration route (systemic, inhaled and topic), using two variables: (i) quartiles of the number of dispensings, and (ii) time of use (recent: at least one dispensing in the year before the index date; past: last dispensing occurring more than one year before the index date; non-use: no use within the preceding three years). Corticosteroids dispensing was identified using ATC codes (**Supplementary Data**, **Table S1**).

### 2.5 Exposure

Exposure to statins was measured through dispensings in the five years prior to the index using ATC codes (**Supplementary Data**, **Table S2**). A six-month lag-time before the index date was used to censor exposure measurement, in order to minimize protopathic bias (related to the possible time between the occurrence of the first AI symptoms and the diagnosis date or drug initiation date) or detection bias (related to an increase of diagnostic procedures in the months before the AI diagnosis date and that could have potentially led to prescribe statins).

The exposure to statins was measured in four different ways: (i) statin use (≥ one dispensing), (ii) statin history of use (new users: first dispensing within the 6 months before lag-time period; current users: first dispensing more than six months before lag-time period and last dispensing within the six months before lag-time period; past users: last dispensing more than six months before lag-time period), (iii) statin cumulative use (total dose dispensed before lag-time period converted and expressed in cumulative years of use by dividing it by the Defined Daily Dose (DDD) of the drug), and (iv) intensity of the last dispensed statin; the reference being always no statin dispensing before lag-time period.

### 2.6 Statistical analyses

We used conditional logistic regression models to compute the odds of AI associated with statin exposure (odds ratios OR and corresponding 95% confidence intervals, 95% CI). Conditional logistic regression models were adjusted for fibrate and ezetimibe use. In addition, statistical models were adjusted for deciles of a disease risk score (DRS) to balance the treatment groups for all potential confounding factors that could alter risk estimation (16). The DRS was estimated by a logistic regression model in all subjects who were never exposed to statins during their follow-up. Values of covariates included in the DRS were measured in the two years before the index date. Potential confounding factors are presented in details in **Supplementary Data**, **Table S3**. The standardized difference between cases and controls was computed for each covariate used as an independent variable in the DRS estimation. A maximal value of 0.1 for standardized difference was considered as satisfying, covariates with a higher value were excluded from the DRS and included into the final model for adjustment.

Secondary statistical analyses consisted in stratifications according to sex, age classes, qualitative use of corticosteroids (non-use vs. at least one dispensing within the three years before index date). Sensitivity analyses were performed to study lag-time variation (assessment with a three-month period and without lag-time), and impact of hospital stay length, by adjusting models for the existence of hospital stays of at least three months within the three years preceding the index date (to take into account periods of hospitalizations when drug exposure could not be identified). Finally, low-dose aspirin (ATC code B01AC06) was used as a negative exposure, this drug having similar indications to statins in cardiovascular prevention.

All analyses were performed using SAS Enterprise Guide 9.4 (SAS Institute, Inc., Cary, NC).

### 2.7 Ethics

By agreement of the French Data Protection Supervisory Authority (*Commission Nationale de l’Informatique et des Libertés*), neither ethics committee approval nor informed consent were required for this observational study based on anonymized French medico-administrative database.

## 3 Results

**Figure 1:**
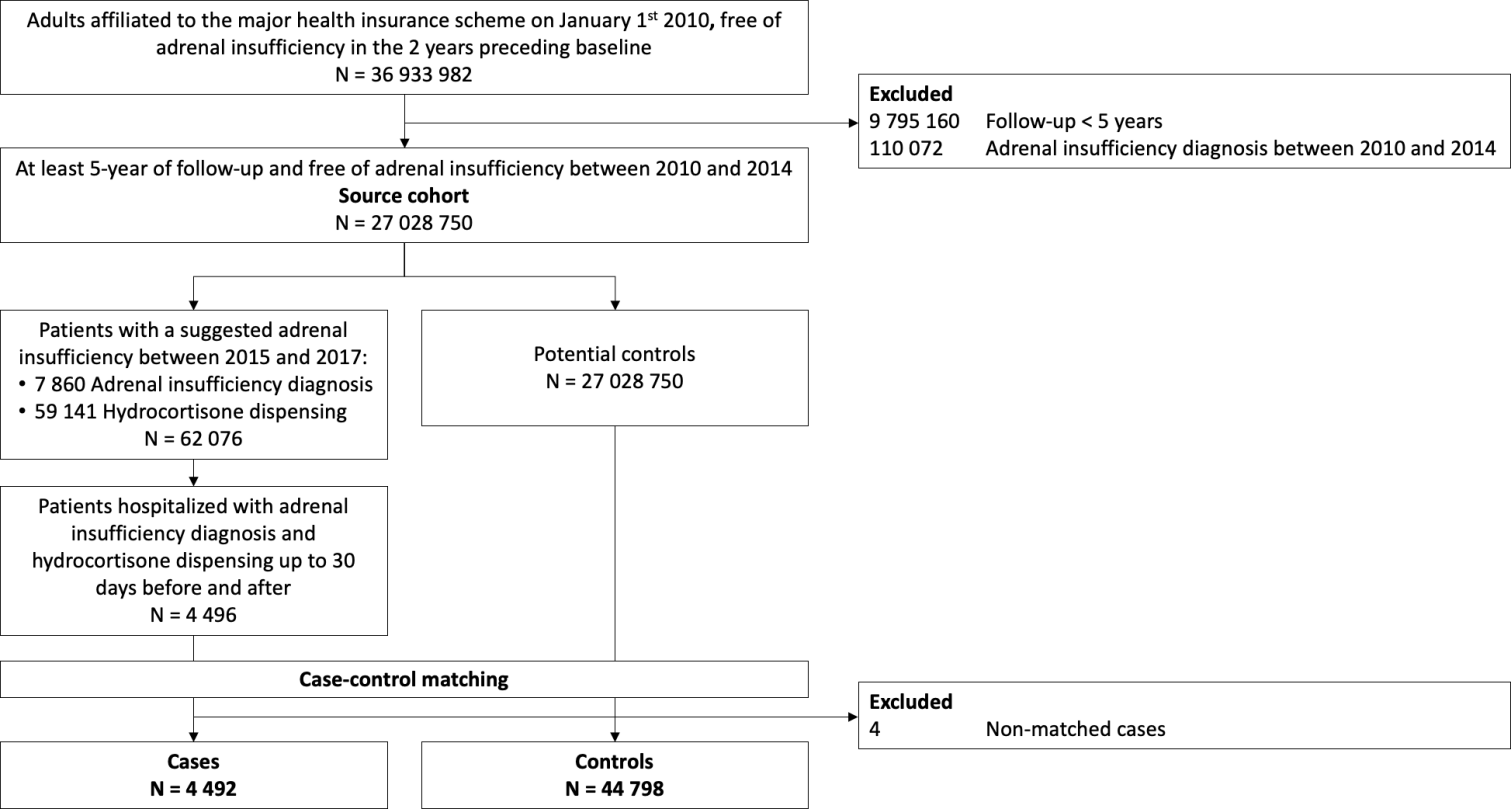
Flow-chart of eligible participants included in study population.

The source cohort included 27 028 250 participants eligible for case and control selection. A total of 4 496 AI cases were identified, of which 4 492 cases were finally matched to 44 798 controls (**Figure 1**). Participants included in the analyses had a median age of 66 years (IQR 52-78), and were mostly women (58.2%). Around 75% had at least one dispensing of systemic corticosteroids or of topical corticosteroids within the three years prior to index date and 40% of inhaled corticosteroids.

Cases appeared to present with a higher burden of comorbidities and a higher use of drugs, potentially confounders in the association between statins and adrenal insufficiency (**Table 1**).

**Table 1:** Characteristics of included cases and controls.

	Cases N = 4 492	Controls N = 44 798
**Age at cohort entry (years),** median (IQR)	66 (52 – 78)	66 (52 – 78)
**Sex - men,** n (%)	1 880 (41.8)	18 732 (41.8)
*Systemic corticosteroids use, n (%)*		
No dispensing	1 097 (24.4)	10 961 (24.5)
Q1: 1-2 dispensings	971 (21.6)	9 709 (21.7)
Q2: 3-6 dispensings	765 (17.0)	7 650 (17.1)
Q3: 7-19 dispensings	828 (18.4)	8 251 (18.4)
Q4: > 19 dispensings	831 (18.5)	8 227 (18.4)
Recent use	2 706 (60.2)	27 001 (60.3)
Past use	689 (15.3)	6 836 (15.3)
*Topical corticosteroids use, n (%)*		
No dispensing	1 110 (24.7)	11 092 (24.8)
Q1: 1-2 dispensings	1 338 (29.8)	13 360 (29.8)
Q2: 3 dispensings	409 (9.1)	4 072 (9.1)
Q3: 4-7 dispensings	833 (18.5)	8 293 (18.5)
Q4: > 7 dispensings	802 (17.8)	7 981 (17.8)
Recent use	2 276 (50.7)	22 714 (50.7)
Past use	1 106 (24.6)	10 992 (24.5)
*Inhaled corticosteroids use, n (%)*		
No dispensing	2 758 (61.4)	27 570 (61.5)
Q1: 1 dispensing	464 (10.3)	4 628 (10.3)
Q2: 2-5 dispensings	417 (9.3)	4 149 (9.3)
Q3: 6-24 dispensings	425 (9.5)	4 205 (9.4)
Q4: > 24 dispensings	428 (9.5)	4 246 (9.5)
Recent use	1 277 (28.4)	12 698 (28.3)
Past use	457 (10.2)	4 530 (10.1)
*Social deprivation index (quintiles), n (%)*		
1 (least deprivation)	822 (18.8)	7 915 (18.2)
2	780 (17.8)	8 147 (18.7)
3	872 (20.0)	8 981 (20.6)
4	942 (21.6)	9 088 (20.8)
5 (highest deprivation)	951 (21.8)	9 401 (21.6)
Missing	125	1 266
*Number of different drugs reimbursed, n (%)*		
Q1: 0-15 drugs	596 (13.3)	12 782 (28.5)
Q2: 16-23 drugs	826 (18.4)	11 356 (25.3)
Q3: 24-33 drugs	1 206 (26.8)	10 935 (24.4)
Q4: >33 drugs	1 864 (41.5)	9 725 (21.7)
*Number of consultations or medical visits, n (%)*		
Q1: 0-100 consultations or medical visits	427 (9.5)	11 901 (26.6)
Q2: 101-176 consultations or medical visits	765 (17.0)	11 566 (25.8)
Q3: 177-297 consultations or medical visits	1 255 (27.9)	11 085 (24.7)
Q4: >297 consultations or medical visits	2 045 (45.5)	10 246 (22.9)
**Alcohol use disorder,** n (%)	320 (7.1)	996 (2.2)
**Tobacco use disorders,** n (%)	1 125 (25.0)	6 432 (14.4)
**Acute coronary syndrome,** n (%)	172 (3.8)	880 (2.0)
**Antiplatelet agents,** n (%)	1 397 (31.1)	10 190 (22.7)
**Anticoagulants,** n (%)	1 386 (30.8)	8 399 (18.7)
**Antihypertensive drugs,** n (%)	2 948 (65.6)	23 894 (53.3)
**Fibrates**, n (%)	302 (6.7)	3 161 (7.1)
**Ezetimibe**, n (%)	258 (5.7)	2 106 (4.7)
**Diabetes,** n (%)	1 152 (25.6)	5 956 (13.3)
**Radiotherapy, local tumors and related procedures that can lead to a secondary adrenal insufficiency,** n (%)	425 (9.5)	645 (1.4)
**CIRCI (critical illness-related corticosteroid insufficiency),** n (%)	796 (17.7)	2 488 (5.5)
**Infections,** n (%)	352 (7.8)	783 (1.7)
**CYP3A4 inducers,** n (%)	165 (3.7)	810 (1.8)
**CYP3A4 inhibitors,** n (%)	2 560 (57.0)	21 724 (48.5)
**Aromatase inhibitors,** n (%)	58 (1.3)	370 (0.8)
**Tyrosine kinase inhibitors,** n (%)	16 (0.4)	13 (0.0)
**Retinoids,** n (%)	57 (1.3)	466 (1.0)
**Antipsychotics,** n (%)	2 727 (60.7)	20 630 (46.0)
**Antidepressants,** n (%)	1 606 (35.7)	10 672 (23.8)
**Interferon or ribavarin,** n (%)	7 (0.2)	29 (0.1)
**Immunotherapy,** n (%)	56 (1.2)	10 (0.0)

Given the very low frequency of tyrosine kinase inhibitors, interferon or ribavirin and immunotherapy (less than 0.1% of controls), these covariates were not included in the DRS. Among the remaining covariates, all showed standardized differences lower than 0.1, except the covariate “radiotherapy, local tumors and related procedures that can lead to a secondary adrenal insufficiency”. This covariate was, as a result, excluded from the DRS and included into the final model for adjustment. Validation information about DRS is detailed in **Supplementary Data**, **Figure S1** and **Table S4**.

Analyses showed no association between statin use and the risk of adrenal insufficiency after adjustment for potential confounders. This absence of association was found regardless of the definition of statin exposure used (statin use, statin history of use, statin cumulative use, or intensity of last statin dispensed). A non-significant increase in AI risk was observed with a cumulative use of]0-1] year (OR 1.09, 95%CI 0.97; 1.27) and for new users (OR 1.12, 95%CI 0.81; 1.55) compared with non-users of statin (**Table 2**). Stratified analyses on sex, age or corticosteroid use did not show any association in some subgroups of patients (**Table 3**).

**Table 2:** Results of the analyses assessing the association between statin exposure and risk of adrenal insufficiency.

	Cases (N = 4 492) n (%)	Controls (N = 44 798) n (%)	Crude OR (95%CI)	Adjusted OR (95%CI) ^a^
Statin use				
No dispensing	2 754 (61.3)	30 174 (67.4)	1.00	1.00
≥1 dispensing	1 738 (38.7)	14 624 (32.6)	1.37 (1.28; 1.47)	0.98 (0.90; 1.05)
Statin history of use				
Non users	2 754 (61.3)	30 174 (67.4)	1.00	1.00
New users	50 (1.1)	336 (0.7)	1.69 (1.25; 2.28)	1.12 (0.81; 1.55)
Current users	1 193 (26.6)	10 125 (22.6)	1.36 (1.26; 1.47)	0.97 (0.89; 1.06)
Past users	495 (11.0)	4 163 (9.3)	1.36 (1.23; 1.51)	0.98 (0.87; 1.09)
Statin cumulative exposure				
0 year	2 754 (61.3)	30 174 (67.4)	1.00	1.00
]0-1] year	458 (10.2)	3 619 (8.1)	1.44 (1.29; 1.60)	1.09 (0.97; 1.22)
]1-2] years	299 (6.7)	2 665 (5.9)	1.29 (1.13; 1.47)	0.95 (0.83; 1.09)
]2-4] years	556 (12.4)	4 905 (10.9)	1.31 (1.19; 1,45)	0.95 (0.85; 1.06)
> 4 years	425 (9.5)	3 435 (7.7)	1.43 (1.28; 1.60)	0.90 (0.79; 1.02)
Intensity of the last dispensed statin				
No dispensing	2 754 (61.3)	30 174 (67.4)	1.00	1.00
Low	249 (5.5)	2 634 (5.9)	1.09 (0.95; 1.25)	0.84 (0.73; 0.98)
Middle	1 029 (22.9)	8 666 (19.3)	1.37 (1.26; 1.48)	1.01 (0.93; 1.11)
High	460 (10.2)	3 324 (7.4)	1.59 (1.43; 1.78)	0.99 (0.88; 1.12)

a The models were adjusted for fibrate use, ezetimibe use, DRS deciles, and a variable grouping radiotherapy use, local tumors, and procedures that could lead to secondary adrenal insufficiency.

**Table 3:** Results of secondary analyses assessing the association between statin use (at least one statin dispensing vs. no dispensing) and risk of adrenal insufficiency stratified by sex, age and corticosteroid use.

	Cases (N = 4 492) n (%)	Controls (N = 44 798) n (%)	Crude OR (95%CI)	Adjusted OR (95%CI) ^a^
Stratification by sex				
Women	872 (33.4)	7 118 (27.3)	1.41 (1.28; 1.55)	1.03 (0.92; 1.14)
Men	866 (46.1)	7 506 (40.1)	1.32 (1.19; 1.47)	0.92 (0.82; 1.03)
Stratification by age				
≤50 years	101 (10.3)	499 (5.1)	2.20 (1.74; 2.78)	1.04 (0.77; 1.40)
51-65 years	484 (38.8)	3 455 (27.7)	1.70 (1.50; 1.92)	1.12 (0.97; 1.29)
66-80 years	724 (53.3)	6 347 (46.8)	1.30 (1.16; 1.46)	0.97 (0.86; 1.10)
>80 years	429 (47.4)	4 323 (47.9)	0.98 (0.85; 1.13)	0.85 (0.74; 0.99)
Stratification by corticosteroid use				
No corticosteroid dispensing	115 (31.9)	869 (24.1)	1.60 (1.23; 2.08)	0.97 (0.71; 1.33)
≥1 dispensing corticosteroid	1 623 (39.3)	13 755 (33.4)	1.35 (1.26; 1.46)	0.97 (0.90; 1.06)

a Models were adjusted for fibrate use, ezetimibe use, DRS deciles, and a variable grouping radiotherapy use, local tumors, and procedures that could lead to secondary adrenal insufficiency.

The results of sensitivity analyses assessing the impact of lag-time length variation and hospital stays longer than three weeks were consistent with those of the main analyses (**Supplementary Data**, **Table S5**). The negative exposure analysis using low-dose aspirin as exposure confirmed also our negative results (**Supplementary Data**, **Table S6**).

## 4 Discussion

We found no relevant association between statin exposure and AI risk after multivariate adjustment for potential confounders and regardless of statin definitions of exposure we used. This result is consistent with those of a recent study describing drug-induced AI using pharmacovigilance reports (17). Our results showed a non-significant increase in AI occurrence for new users of statins and for cumulative use of statins lower than one year. This risk, if any, seems to be very limited given the risk estimates not exceeding 1.15. This non-significant association observed when treatment with statin was initiated may be due to a residual detection bias rather than to an effect of statin on AI. From a biological and physiological viewpoint, a cumulative dose-effect would rather have been expected.

London et al. had shown in their study of 14 patients that a single treatment with a lipophilic statin reduced the production of some glucocorticoid precursors (12). This effect could have a clinical impact but it is possible that a compensatory phenomena allow glucocorticoid synthesis to be maintained or regulated.

Our study presents with strengths and limitations common to all studied performed from medico-administrative databases. As for their strengths, the nationwide healthcare database of the French population is frequently used for safety studies owing to their interest in terms of data exhaustiveness, and representativeness of the population (15, 18–20). Our study hence focused on data relating to beneficiaries of the major health insurance scheme, which concern about 80% of the French population. As for their limitations, this database only informs on reimbursed dispensings for prescribed drugs, which is only a proxy of patients’ exact use. As for the strengths specific to our study, the multivariate adjustment including a DRS succeeded in considering for a big part of confounding as it removed the association initially found with the crude analysis. In addition, all performed sensitivity analyses were consistent, including the analyses using low-dose aspirin as negative tracer exposure which eliminates a possible indication bias. As for the limitations of our study, the way we identified AI has not been validated elsewhere, and did not used steroid levels. However, it has been internally discussed with clinical experts in endocrinology and hospital coding. In addition, other studies on the same topic have used similar identification algorithm (AI ICD codes associated with hydrocortisone use) to define AI in claim databases (21, 22). Finally, our study only involved adult subjects, whereas in heterozygous familial hypercholesterolemia statin treatment can be initiated at a very young age. The impact of such treatment at this young age on steroid hormone synthesis could not therefore be studied here and would require further investigations, even if clinical trials conducted in this population have been reassuring (23, 24).

To the best of our knowledge, our study is the first to investigate the risk of AI associated with statin use in nationwide data. Our results do not indicate an increase in the occurrence of AI associated with statin use, either at initiation, with high cumulative use or high intensity treatment. The risk of AI related to statin exposure, if any, seems to be very limited and does not compromise statin treatment. Further studies could be carried out to assess the impact of these drugs on steroid hormone synthesis in more at-risk population or young patients.

## Data availability statement

The data analyzed in this study is subject to the following licenses/restrictions: French law to access SNDS. Requests to access these datasets should be directed to https://www.health-data-hub.fr.

## Author contributions

SM: Conceptualization, Methodology, Writing – original draft, Writing – review & editing. AJ: Conceptualization, Formal Analysis, Methodology, Writing – review & editing. AP: Conceptualization, Methodology, Supervision, Writing – review & editing. TD: Conceptualization, Methodology, Writing – review & editing. LD: Conceptualization, Methodology, Writing – review & editing. JB: Conceptualization, Methodology, Supervision, Writing – review & editing.
