# Supplementary material for: Statin treatment is not associated with an increased risk of adrenal insufficiency in real-world setting
Source: Front Endocrinol (Lausanne). 2023 Sep 25;14:1254221. doi: 10.3389/fendo.2023.1254221 (PMC10561645; doi:10.3389/fendo.2023.1254221)
Supplement: Supplementary file 1 [file DataSheet_1.docx]

Supplementary Material

Statins and the risk of adrenal insufficiency: a nested case-control study

Sandy Maumus-Robert, Ana Jarne-Munoz, Antoine Pariente, Thomas Duroux, Lise Duranteau, Julien Bezin*

*** Correspondence:** Julien Bezin: [julien.bezin@u-bordeaux.fr](mailto:julien.bezin@u-bordeaux.fr)

**Table S1. Codes used for the identification of corticosteroids**

| **Administration route** | **ATC codes** |
| --- | --- |
| **Systemic** | H02AA02 H02AB01 H02AB02 H02AB04 H02AB06 H02AB07 H02AB08 H02AB13 H02AB17 R06AB52 |
| **Inhaled** | R03AK06 R03AK07 R03AK08 R03AK10 R03AK11 R03AL08 R03AL09 R03BA01 R03BA02 R03BA03 R03BA04 R03BA05 R03BA06 R03BA07 R03BA08 R03BA09 |
| **Topical** | A01AC A07EA02 A07EA04 A07EA06 C05AA D07 R01AD S01BA S01CA S02B S02C |

**Table S2. Codes used for the identification of statins**

|  | **ATC code** | **DDD** | **Dosage intensity** |
| --- | --- | --- | --- |
| **Simvastatin** | C10AA01 | 30 mg | Low: 10 mg  Moderate: 20-40 mg |
| **Pravastatin** | C10AA03 | 30 mg | Low: 10-20 mg  Moderate: 40 mg |
| **Fluvastatin** | C10AA04 | 60 mg | Low: 20-40 mg  Moderate: 80 mg |
| **Atorvastatin** | C10AA05 | 20 mg | Moderate: 10 mg  High: 20-80 mg |
| **Rosuvastatin** | C10AA07 | 10 mg | Moderate: 5 mg  High: 10-20 mg |
| **Simvastatin and ezetimibe** | C10BA02 | 1 unit | Moderate: 20-40 mg |
| **Atorvastatin and ezetimibe** | C10BA05 | 1 unit | Moderate: 10 mg  High: 20-80 mg |
| **Pravastatin and aspirin** | C10BX02 | 1 unit | Moderate: 40 mg |
| **Atorvastatin and amlodipine** | C10BX03 | 1 unit | Moderate: 10 mg |

Abbreviation: ATC, Anatomical Therapeutic Chemical classification system; DDD, Defined Daily Dose

**Table S3. Codes used for the identification of covariates**

|  | **Identification** |
| --- | --- |
| **Social deprivation index** | Fdep99 score based on living town |
| **Number of medical visits/consultations** | In classes, according to distribution quartiles |
| **Number of different drugs reimbursed** | In classes, according to distribution quartiles |
| **Alcohol use disorders** | *Ad hoc* algorithm available upon request |
| **Tobacco use disorders** | *Ad hoc* algorithm available upon request |
| **Acute coronary syndrome** | ICD-10 codes: I200, I21, I22, I23, I24  French procedure codes: DDAF001 DDAF003 DDAF004 DDAF006-DDAF010 YYYY082 DDPF002 DDMA003-DDMA009 DDMA011-DDMA013 DDMA015-DDMA038 ENFA003 EPFA006 DDAA002 DDFF001 DDFF002 |
| **Antihypertensive drugs** | ATC codes: C03, C07, C08, C09, C02AC, C02CA, C02AB, C02DC, C02L |
| **Anticoagulants** | ATC codes: B01AA, B01AB, B01AE, B01AF, B01AX05 |
| **Antiplatelet agents** | ATC codes: B01AC |
| **Fibrates** | ATC codes: C10AB02 C10AB04 C10AB05 C10AB08 |
| **Ezetimibe** | ATC codes: C10AX09 C10BA02 C10BA05 |
| **Diabetes** | ICD-10 codes: E10, E11, E12-14  ATC codes: A10A, A10B |
| **Infections (bacterial:** tuberculosis, Haemophilus influenzae, Treponema pallidum, meningococcus, streptococcus, staphylococcus aureus; **parasitic:** *Trypanosoma brucei*; v**iral:** HIV, CMV, Herpes Simplex Virus; **fungal:** histoplasmosis, cryptococcosis, coccidioidomycosis, blastomycosis, paracoccidioidomycosis) | ICD-10 codes: A15-A19, A492, B963, G00, J14, J201, P236, A413, A50-A52, A39, A40, A491, I301 and {B950, B951, B952, B953, B954, B955, B956}, G002, J020, J13, J153, J154, J202, M002, P233, P236, Z223, A410, A490, B957, B958, G003, J152, M000, P232, B56, B202, B25, A60, B00, B39, B45, B38, B40, B41,  B20-B24, F024, Z21  ATC codes: J05AF01 J05AF02 J05AF04 J05AF05 (except French drug codes: 3400935196712, 3400935196941), J05AF06 J05AF07 J05AF09, J05AG01 J05AG03 J05AG04 J05AG05 J05AG06, J05AJ01 J05AJ03 J05AJ04 J05AR01 J05AR02 J05AR03 J05AR04 J05AR06 J05AR08 J05AR09 J05AR10 J05AR13 J05AR17 J05AR18 J05AR19 J05AR20 J05AR21 J05AR24 J05AR25, J05AE01 J05AE02 J05AE03 J05AE04 J05AE05 J05AE07 J05AE08 J05AE09 J05AE10, J05AX07 J05AX09 J05AX23) |
| **CIRCI (critical illness-related corticosteroid insufficiency)** | ICD-10 codes: J13-J18, A021, A267, A227, A40, A41, B007, B377, R572, J80, I46, S02, S06, S09, F072, G443, G913, T905, T794, T20-T32  French procedure codes: DAGA001, DBFA002, DAFA003, DAFA006, DAFA008, DBFA001, DAMA900, DASA006, DBPA002, DBPA004, DBPA006, DBPA007, DBMA002, DBMA003, DBMA008, DBMA011, DBMA012, DBKA001, DBKA002, DBKA003, DBKA004, DBKA005, DBKA006, DBKA007, DBKA008, DBKA009, DBKA010, DBKA011, DBKA012, DBMA005, DBMA006, DBMA007, DBMA009, DBMA010  DBMA013, DZEA001, DZEA003, DZEA004 |
| **Radiotherapy, local tumors and related procedures that can lead to a secondary adrenal insufficiency** | ICD-10 codes: D430, D4440, C751, C793, D352, D443  French procedure codes: ZZMK014 ZZMK002 ZZMK013 ZZMK026 ZZMK028 ZZMK001 ZZMK017 ZZMK016 ZZMK011 ZZMK018 ZZMK024 ZZMP001QZMP003 AGMP001 ZZMP018 ZZMP012 ZZMP016 ZZMP013 ZZMK019 ZZMK027 ZZMK022 ZZMK020 ZZMK025 ZZNL061 ZZNL053 ZZNL064 ZZNL048 ZZNL065 ZZNL062 ZZNL063 ZZNL050 ZZNL054 ZZNL051 ZZNL058 ZZNL052 ZZNL059 ZZNL060 ZANL001 ZZNL049 ZZNL055 AZNL001 ZZNL045 ZZNL046 ZZNL047 QZNL001 ZZNL066 ZZMK006 ZZMK012 ZZMK003 ZZMK004 ZZMK005 ZZMK007 ZZMK010 ZZMK015 ZZMP008 ZZMK900 ZZMP010 ZZMP900 AZMP001 ZZMP011 ZZMK021 ZZMK023 ZZNL020 ZZNL021 ZZNL023 ZZNL024 ZZNL025 ZZNL026 ZZNL027 ZZNL028 ZZNL030 ZZNL031 ZZNL033 ZZNL034 ZZNL036 ZZNL037 ZZNL039 ZZNL040 ZZNL042 ZZNL043 ZZNL900 ZZNL902 ZZNL903 ZZNL904 ZZNL905 ZZNL906 AANL001 AANL002, KAFA001, KAFA002, KAFE900, KANB001, AAFA002, AAFA005, AAFA001, AAFA003, AAFA008, ABFA008, ABFA009, ABFA010, ACFA001, ACFA002, ACFA003, ACFA004, ACFA005, ACFA006, ACFA007, ACFA008, ACFA009, ACFA010, ACFA011, ACFA012, ACFA013, ACFA014, ACFA015, ACFA016, ACFA018, ACFA019, ACFA020, ACFA022, ACFA023, ACFA024, ACFA025, ACFA026, ACFA027, ACFA028, ACFA029, ABFA002, ABFA005, ABFA006, ABFC001, ABFC002, GCFA001, GCFA002, GCFA003, GCFA004, GCFA005, GCFA006, GCFA007, GCFD001, GCFD002 |
| **CYP3A4 inducers** (bosentan, carbamazepine, cyclophosphamide, efavirenz, elvitegravir, enzalutamide, etravirine, felbamate, ifosfamide, meprobamate, metamizole, modafinil, nevirapine, oxcarbazepine, perampanel, phenobarbital, phenytoin, primidone, rifabutin, rifampicin, topiramate, vinblastine) | ATC codes: C02KX01 N03AF01 L01AA01, J05AG03 J05AR06 J05AR11 J05AJ02 J05AR18 J05AR09 L02BB04 J05AG04 N03AX10 L01AA06 N05BC01 N05BC51 N05CX01 N02BB02 N02BB52 N02BB72 N06BA13 N06BA07 J05AG01 J05AR07 J05AR05 N03AF02 N03AX22 N03AA02 N03AB02 N03AB52 N03AA03 J04AB04 J04AB02 J04AM02 J04AM07 J04AM05 J04AM06 N03AX11 L01CA01  French drug codes: 9426947 9426947 9435320 9430386 9431233 9433114 9436087 9440166 9308679 9396442 9396442 9396442 9416713 9404331 9404331 9404331 9410188 9410188 9410188 9437980 9212028 9212011 9201065 9201065 9240562 9410188 9410188 9437980 9437980 9437980 9396442 9396442 9396442 9396442 9404331 9404331 9404331 9404331 9404331 9404331 |
| **CYP3A4 inhibitors** (fusidic acid, amiodarone, amprenavir, atazanavir, boceprevir, ciclosporin, cimetidine, ciprofloxacin, clarithromycin, clobazam, cobicistat, darunavir, dasatinib, delavirdine, desogestrel, diltiazem, dronedarone, doxycycline, erythromycin, ethinylestradiol, fluconazole, gestodene, grazoprevir, imatinib, indinavir, isoniazid, itraconazole, josamycin, ketoconazole, lopinavir, miconazole, nelfinavir, nifedipine, nitrendipine, nilotinib, posaconazole, quetiapine, ritonavir, roxithromycin, saquinavir, simeprevir, sorafenib, telithromycin, verapamil, voriconazole) | ATC codes: D06AX01 D09AA02 J01XC01 S01AA13 01BD01 J05AE05 J05AR15, J05AR23, J05AE08 J05AP03 L04AD01, S01XA18 A02BA01 A02BA51 J01MA02 S01AE03 S02AA15 S03AA07 J01RA10 J01RA11 J01RA12 J01FA09 A02BD06 A02BD07 A02BD09 A02BD05 A02BD04 A02BD11 A02BD12 A02BD14 N05BA09 J05AR09 J05AR14 J05AR15 J05AR18 J05AR22 V03AX03 J05AE10 J05AR26 L01EA02 J05AG02 L01EA02 J05AG02 G03AC09 G03FB10 G03AA09 G03AB05 C05AE03 C08DB01 A01AB22 J01AA02 D10AF02 J01FA01 S01AA17 D10AF52 G03AA15 G03AB07 G03AA09 G03AB05 G03AA16 G03AA12 G03CA01 L02AA03 G03AA01 G03AA10 G03AB06 G03AA07 G03AB03 G03AA03 G03AB02 G03AA08 G03AA04 G03AB01 G03AA13 G03AA05 G03AB04 G03AA11 G03AB09 G03AA06 G03AA02 J01RA07 D01AC15 J02AC01 J05AP11 J05AP54 L01EA01 J05AE02 J04AM03 J04AC01 J04AC51 J04AM08 J04AM01 J04AM04 J04AM02 J04AM07 J04AM05 J04AM06 J02AC02 J01FA07 D01AC08 G01AF11 H02CA03 J02AB02 J05AR10 A01AB09 A07AC01 D01AC02 G01AF04 J02AB01 S02AA13 D01AC52 J05AE04 C07FB03 C08CA05 C08GA01 C08CA55 C08CA08 C09BB06 L01EA03 J02AC04 N05AH04 J05AE03, J05AR10, J05AR23, J05AR26, J05AP52, J05AP53 J01FA06 J05AE01 J05AP05 L01EX02 J01FA15 C08DA01 C08DA51 C09BB10 J02AC03  French drug codes: 9446163 9446140 9446157 9444075 9441616 9441622 9450816 9450816 9450839 9450839 9444075 9444075 9441616 9441616 9441622 9441622 9449888 9449871 9448529 9448512 9448423 9448423 9448423 9448452 9448452 9448452 9448446 9448446 9450822 9450822 9451678 9258697 9258705 9312907 9312907 9446163 9446163 9450822 9450839 9448446 9448446 9448446 9448452 9448452 9448452 9211980 9211980 9180009 9180015 9179963 9211980 9179992 9422694 9422688 9446306 9446298 9082092 9180009 9180009 9180015 9180015 9179963 9179963 9179992 9397217 9000741 9000741 9000741 9000741 9397217 9397217 9000909 9288528 9288528 9396614 9393455 9413347 9393461 9391025 9441645 9441645 9441651 9441651 9441668 9441668 9443704 9443704 9443710 9443710 9435981 9435981 9435981 9435981 9435981 9435981 9435998 9435998 9445229 9445229 9445229 9445229 9445235 9445235 9445235 9445241 9445229 9445235 9441125 9441125 9441125 9441125 9441125 9441125 9441131 9441131 9441131 9441131 9441131 9441148 9441148 9441148 9441148 9447234 9447240 9447257 9440976 9440982 9441007 9447062 9447079 9446097 9446105 9446128 9452399 9452407 9328050 9328067 9339183 9339177 9443710 9445235 9445241 9445241 9445241 9441125 9441131 9441131 9441148 9441148 9441148 9447257 9446128 9447257 9442343 9445910 9445904 9445956 9445956 9445933 9445933 9445927 9445927 9450845 9450851 9450851 9450868 9450874 9450874 9450880 9450880 9456486 9456486 9456463 9456463 9456463 9456517 9456517 9456517 9456500 9456500 9456500 9456492 9456492 9456492 9456428 9456411 9456457 9456440 9456434 9456440 9456434 9456457 9457089 9457089 9457095 9457095 9457103 9457103 9457103 9457066 9457066 9457072 9457072 9000547 9000547 9000547 9000547 9000548 9000548 9000548 9000548 9000549 9000549 9000549 9000549 9000545 9000545 9000546 9000546 9000547 9000548 9000549 9000546 9000545 9001200 9001200 9001200 9001201 9001201 9001201 9001203 9001203 9001203 9001198 9001198 9001198 9293311 9293311 9293311 9293328 9293328 9293328 9293506 9293506 9293506 9326223 9326223 9372714 9372714 9450868 9450874 9450880 9450845 9450845 9450851 9450851 9456428 9456411 9000545 9000546 9450880 9219993 9418008 9411414 9415292 9433278 9352410 9366116 9405750 9405750 9441674 9441680 9438910 9229158 9286995 9286995 9312072 9286995 9181664 9275891 9401108 9401108 9446140 9446140 9446157 9446157 9446163 |
| **Aromatase inhibitors** | ATC codes: L02BG |
| **Tyrosine kinase inhibitors** (sunitinib, cabozantinib) | French drug codes: 3400937626507 3400937626675 3400937626736 3400938210224 3400938210392 3400938210453 3400930073520 3400930073537 3400930073544 3400958900822 3400958900839 3400958900846 9284915 9284921 9284938 9284915 9284921 9284938 9420005 9420011 9420028 9420005 9420011 9420028 |
| **Retinoïds** | ATC codes: D05BB, D10AD, D10BA |
| **Antipsychotics** | ATC codes: N05 |
| **Antidepressants** | ATC codes: N06 |
| **Interferon or ribavirin** | ATC codes: J05AP01, L03AB04, L03AB05, L03AB11  French drug codes: 3400921695656 3400921695717 3400921695885 3400926709280 3400926709341 3400926709402 3400926709570 3400927327476 3400927327537 3400927900709 3400927900877 3400927900938 3400927901010 3400927901188 3400927901249 3400927901300 3400935197191 3400935197252 3400935197313 3400936159082 3400936159143 3400936200395 3400936200456 3400936586680 3400937327787 3400937779531 3400937779760 3400941588150 3400941588211 3400941588389 3400941588440 3400941682391 3400941682452 3400941682513 3400941682681 3400941682742 3400941682803 3400941682971 3400941683053 3400941683114 3400941683282 3400941684173 3400941684234 3400941684463 3400941684524 3400941684692 3400941684753 3400941684814 3400959003171 3400959003232 3400930038208 3400930038246 3400930038277 3400935256256 3400935256546 3400935256775 3400935257147 3400935257376 3400935257727 3400934956287 3400935965424 3400935965592 3400935965653 3400921716801 3400921716979 3400921717341 3400921718171 3400926980511 3400935995810 3400935995988 3400935996060 3400935996121 9373381 9373381 9373398 9367587 9367587 9367587 9367587 9408949 9408949 9406413 9406413 9406413 9406413 9406436 9406436 9406436 9208570 9208570 9208570 9250187 9250187 9250187 9250187 9274408 9208570 9298610 9298610 9367587 9367587 9367587 9367587 9373346 9373346 9373346 9373346 9373346 9373346 9373352 9373352 9373352 9373352 9373381 9373381 9373381 9373381 9373398 9373398 9373398 9172487 9172470 9220861 9220884 9220890 9220884 9220884 9220890 9220890 9220861 9220861 9206708 9247110 9247110 9247110 9374682 9374682 9374699 9374699 9395388 9241047 9241047 9241076 9241076 |
| **Immunotherapy** |  |
| Monoclonal antibodies anti-PDL1: Durvalumab (IMFINZI 500 and 120), Avelumab (BAVENCIO), Atezolizumab (TECENTRIQ 1200 mg; TECENTRIQ 840) | ATC codes: L01XC28 L01XC31 L01XC32  French drug codes: 3400955058144 3400955058151 3400958901126 3400958901232 3400958902130 3400959116925 34009550415283 3400958900921 3400958902369 3400959109132 3400955042006 3400955067894 3400958901737 3400958901829 3400958902277 3400959109590 9440545 9440551 9436294 9437939 9440545 9433485 9430736 9424322 9430736 9422518 9431262 9000225 9431262 9451431 9431262 9423179 |
| Monoclonal antibodies anti-PD1: Pembrolizumab, Nivolumab, Cémiplimab, dostarlimab | ATC codes:L01XC18 L01XC17 L01XC33 L01XC40  French drug codes: 3400955006558 3400955024316 3400955005797 3400955005803 3400955055570 3400958902888 3400958902895 3400955067283 3400958901508 3400959000477 3400959001306 3400958902659 3400959003058 9409535 9419723 9409469 9409452 9438979 9409469 9438979 9447470 9447470 9439789 9447470 9000380 |
| Monoclonal antibodies anti-CTLA4: Ipilimumab | ATC codes: L01XC11  French drug codes: 3400958087707 3400958087875 3400958902901 3400958929779 3400958929830 3400959070067 9374050 9374067 9374050 9372217 9321013 9321013 |

Abbreviation: ATC, Anatomical Therapeutic Chemical classification system; ICD-10, International Statistical Classification of Diseases and Related Health Problems 10^th^ revision

**
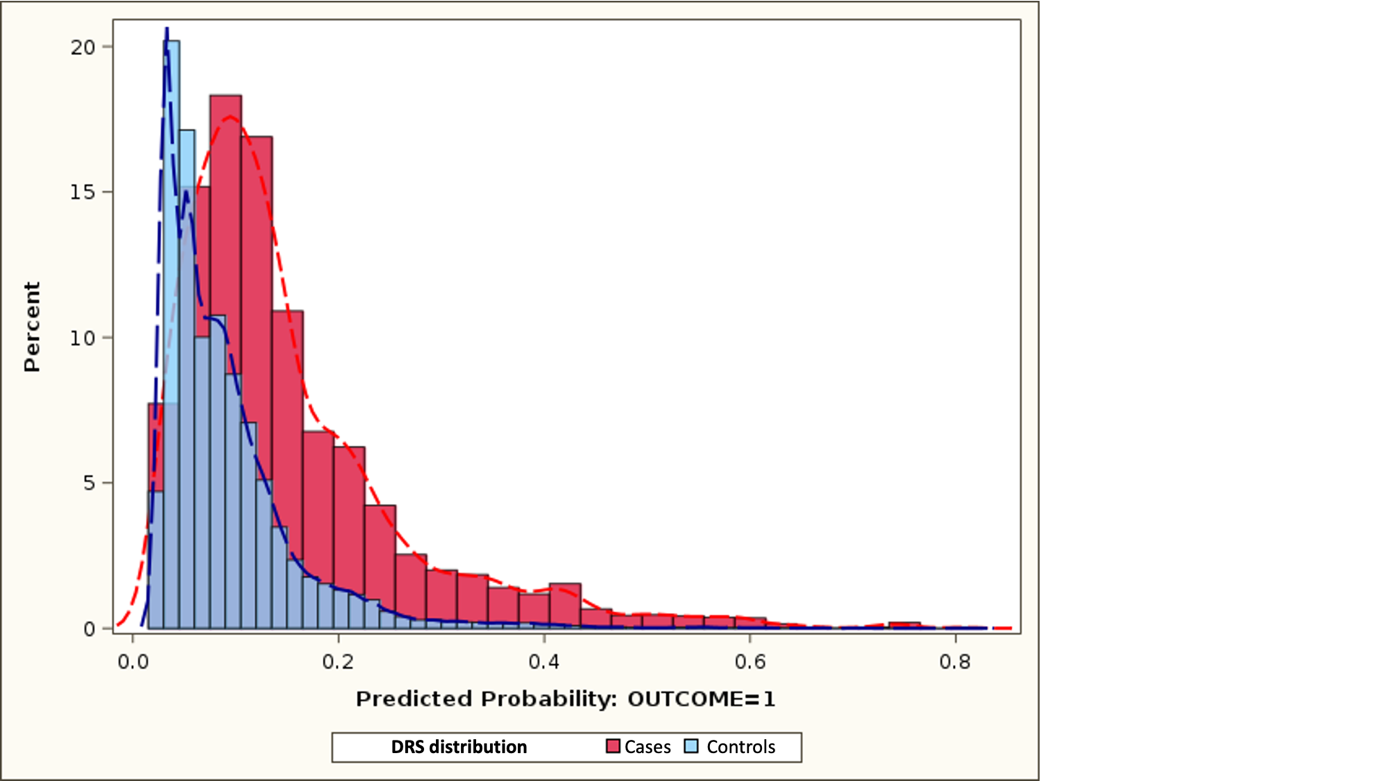
**

**Figure S1. Distribution of disease risk score between case and control groups**

Cases are displayed in red and controls are displayed in blue.

**Table S4. Estimation of standardized deviations of covariates included in the disease risk score**

|  | **Standardized differences** |
| --- | --- |
| **Social deprivation index** |  |
| Q1 | 0.059 |
| Q2 | 0.034 |
| Q3 | 0.054 |
| Q4 | 0.049 |
| Q5 | 0.066 |
| **Alcohol use disorder treatment** | 0.075 |
| **Number of medical visits/consultations** |  |
| Q1 | 0.059 |
| Q2 | 0.052 |
| Q3 | 0.069 |
| Q4 | 0.060 |
| **Number of different drugs reimbursed** |  |
| Q1 | 0.077 |
| Q2 | 0.060 |
| Q3 | 0.056 |
| Q4 | 0.056 |
| **Acute coronary syndrome** | 0.080 |
| **Tobacco use disorder treatment** | 0.059 |
| **Antihypertensive drugs** | 0.042 |
| **Anticoagulants** | 0.059 |
| **Antiplatelet agents** | 0.051 |
| **Diabetes** | 0.065 |
| **Infections** | 0.059 |
| **CIRCI** | 0.097 |
| **CYP3A4 inducers** | 0.042 |
| **CYP3A4 inhibitors** | 0.030 |
| **Aromatase inhibitors** | 0.047 |
| **Retinoïds** | 0.056 |
| **Antipsychotics** | 0.050 |
| **Antidepressants** | 0.059 |

**Table S5. Sensitivity analyses assessing the impact of lag-time length variation and hospital stays longer than three weeks**

|  | **Cases**  **(N = 4 492)**  n (%) | **Controls**  **(N = 44 798)**  n (%) | **Crude OR**  **(95%CI)** | **Adjusted OR (95%CI) *** |
| --- | --- | --- | --- | --- |
| **3-month lag-time period** |  |  |  |  |
| No dispensing | 2 732 (60.8) | 30 009 (67.0) | 1.00 | 1.00 |
| ≥1 dispensing | 1 760 (39.2) | 14 789 (33.0) | 1.38 (1.28-1.48) | 0.98 (0.90-1.05) |
| **No lag-time period** |  |  |  |  |
| No dispensing | 2 703 (60.2) | 29 854 (67.0) | 1.00 | 1.00 |
| ≥1 dispensing | 1 789 (39.8) | 14 944 (33.0) | 1.40 (1.30-1.50) | 0.99 (0.91-1.07) |
| **Adjustment for long hospital stays** |  |  |  |  |
| No dispensing | 2 754 (61.3) | 30 174 (67.4) | 1.00 | 1.00 |
| ≥1 dispensing | 1 738 (38.7) | 14 624 (32.6) | 1.37 (1.28-1.47) | 0.98 (0.91-1.06) |

* The models were adjusted for fibrate use, ezetimibe use, DRS deciles, and a variable grouping radiotherapy use, local tumors, and procedures that could lead to secondary adrenal insufficiency.

**Table S6. Sensitivity analysis assessing the association between low-dose aspirin use and risk of adrenal insufficiency**

|  | **Cases**  **(N = 4 492)**  n (%) | **Controls**  **(N = 44 798)**  n (%) | **Crude OR**  **(95%CI)** | **Adjusted OR (95%CI) *** |
| --- | --- | --- | --- | --- |
| **Low-dose aspirin use** |  |  |  |  |
| No dispensing | 3 135 (69.8) | 34 259 (76.5) | 1.00 | 1.00 |
| ≥1 dispensing | 1 357 (30.2) | 10 539(23.5) | 1.50 (1.39-1.61) | 1.00 (0.92-1.09) |
| **Low-dose aspirin history of use** |  |  |  |  |
| Non users | 3 135 (69.8) | 34 259 (76.5) | 1.00 | 1.00 |
| New users | 77 (1.7) | 518 (1.2) | 1.72 (1.35,2.19) | 1.03 (0.79, 1.34) |
| Current users | 857 (19.1) | 6 666 (14.9) | 1.50 (1.38,1.64) | 1.01 (0.92, 1.11) |
| Past users | 423 (9.4) | 3 355 (7.5) | 1.46 (1.31,1.63) | 0.97 (0.86, 1.10) |

* The models were adjusted for fibrate use, ezetimibe use, DRS deciles, and a variable grouping radiotherapy use, local tumors, and procedures that could lead to secondary adrenal insufficiency.
